# Morphology of Models Manufactured by SLM Technology and the Ti6Al4V Titanium Alloy Designed for Medical Applications

**DOI:** 10.3390/ma14216249

**Published:** 2021-10-21

**Authors:** Damian Gogolewski, Tomasz Kozior, Paweł Zmarzły, Thomas G. Mathia

**Affiliations:** 1 Department of Manufacturing Technology and Metrology, Kielce University of Technology, 25-314 Kielce, Poland; tkozior@tu.kielce.pl (T.K.); pzmarzly@tu.kielce.pl (P.Z.); 2 Laboratoire de Tribologie et Dynamique des Systemes (LTDS)—CNRS Ecole Centrale de Lyon, 69134 Lyon, France; Thomas.Mathia@ec-lyon.fr

**Keywords:** dimples, bumps, surface texture, medicine, additive manufacturing, SLM

## Abstract

This paper presents the results of an experimental study to evaluate the possibility of using SLM additive technology to produce structures with specific surface morphological features. Qualitative and quantitative tests were conducted on samples fabricated by 3D printing from titanium (Ti6Al4V)-powder-based material and analysed in direct relation to the possibility of their use in medicine for the construction of femoral stem and models with a specific degree of porosity predicted by process-control in the self-decision-making 3D printing machine. This paper presents the results of the study, limitations of the method, recommendations that should be used in the design of finished products, and design proposals to support the fabrication process of 3D printers. Furthermore, the study contains an evaluation of how the printing direction affects the formation of certain structures on the printed surface. The research can be used in the development of 3D printing standardization, particularly in the consideration of process control and surface control.

## 1. Introduction

Over the past several years, unimaginable developments have occurred in the chemical composition of materials used in 3D printing technologies. Today, additive technologies enable the fabrication of models from plastic-based materials, metals, and ceramics. The introduction of additives to increase strength, corrosion resistance, heat resistance, and chemical resistance has made 3D printing technologies in the era of Industry 4.0 widely used in the foundries [1,2,3,4], jewellery, aerospace, automotive [5,6,7], defence, medicine [8,9], and pneumatic and hydraulic industries [10]. In addition, materials are now being used that enable fabrication from composite-based materials, particularly in technologies based on fused deposition modelling [11,12]. In connection with the ongoing Industry 4.0 revolution and new opportunities, the topic of 4D printing has seen increased interest, where through the use of smart material and other technologies, such as, for example, electrospinning, it is also possible to build advanced cell structures or composite models [13,14,15].

**Figure 1:**
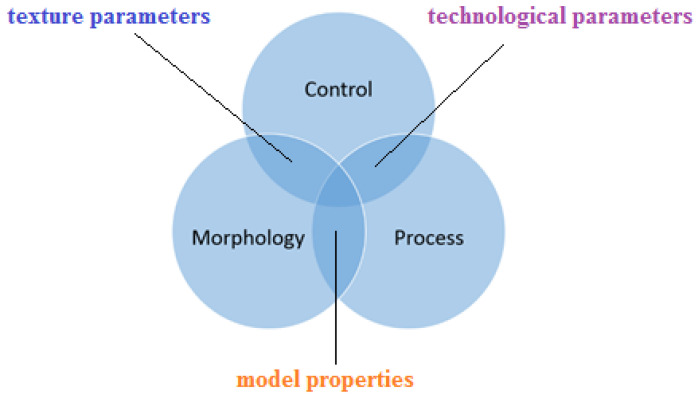
Relationship between surface morphology, control, and process.

It is crucial for 3D printing production of high-quality models expressed by dimensional and shape accuracy, surface texture parameters, mechanical strength, abrasive wear resistance, etc., to consider the so-called process-control [16,17,18,19,20,21,22,23]. Process-control takes into account the analysis of many technological parameters depending on the 3D printing technology, such as: thickness of the fabricated layer, temperature of the build chamber, speed and power of the laser, diameter of the focused beam, printing nozzle diameter, format and accuracy of the digital file before printing, and many other parameters with a direct impact on the quality of the fabricated model. Improper process control is also associated with adverse phenomena, for example, the “orange peel,” occurring with technologies such as selective laser sintering. It also seems that the shape of the fabricated model—in particular, in the case of very small elements with dimensions below one millimetre—can have a very large effect. This shape causes a collision at the cross-section between the technological capabilities of the machine and the geometry we want to obtain. 3D printer software cannot always compensate for certain actions, and sometimes it does not even try to do so due to the impossibility of such compensation. Consequently, for small models or elements of models, a technological problem of fabrication arises, which is particularly evident for 3D models with a specific degree of porosity, which is very challenging in medical applications. Porosity affects the phenomena occurring during tissue growth on bone implants and the adhesion force when coating the models. If the process is controlled to achieve an appropriate degree of porosity in models printed with additive technologies, it is possible to use a finishing treatment called laser ablation [24,25]. The relationship between surface morphology, control, and process is very important, as shown in Figure 1.

As can be seen in the era of Industry 4.0 [26], it is the medical field that is one of the main customers for models fabricated by 3D printing, and the machines used in these technologies are some of the most advanced and accurate. In medicine, we have to distinguish between two main applications of the 3D printing technology, namely, the printing of bone implants, which is particularly prominent in orthopedics, and bio-printing with tissues. In the first case, the process uses conventional 3D printers, and in the second case, sophisticated machines with very high accuracy and biocompatibility of the model-building process are employed.

In orthopedics, 3D printers can fabricate bone implants from biocompatible materials and in forms that are geometrically perfect for a given patient; this is because the 3D printing process can build directly from mathematical CAD models obtained from three-dimensional imaging methods such as computed tomography (CT), magnetic resonance imaging (MRI), 3D scanning, and other methods used to assess the skeletal health of a patient.

In the case of technologies using metal-powder-based materials such as selective laser melting (SLM, direct metal laser sintering), DMLS, laser engineered net shaping (LENS, used for materials such as corrosion-resistant steel), 316L [27], titanium-based steels, maraging steel, cobalt-chrome steel, aluminum alloy, nickel super alloy, tool steels, and multiple other metal-powder-based mixtures [28].

Studies regarding the mechanical properties and accuracy [29,30,31,32] of models fabricated with metal-based powder technologies have been described in multiple research papers, but when it comes to surface porosity measurements of models manufactured with additive technologies, the number of these studies is still insufficient and simulation studies attempting to model the porosity formation process are scarce [33,34,35,36].

Analysis of porous models (orthopedic implants) with biomedical applications are described in [33]. The authors of this study developed 3D models that were characterized by a uniform distribution of very small geometric features representing surface porosity. These models were made using the SLM technology with titanium-based powder. Porosity measurements were performed, and the relationship between the porosity of the CAD model and the actual porosity after printing with 3D printing technology was determined.

An overview of the application possibilities of the 3D printing technology to build models of bone scaffolds and orthopedic implants is provided in [34]. This article mainly analyses laser 3D printing technologies that use metal-powder-based materials. The topological design of porous metallic structures for orthopedic implants has also been extensively described.

Another example of using laser 3D printing technologies to build cylindrical porous samples with diamond unit cells of titanium-based Ti6Al4V powder using Selective Laser Melting (SLM) is described in [35]. The research involved both porosity and strength evaluation of the built models. The paper provides tools for improving fatigue performance of porous models in medical applications. In paper [37], the application of Ti6Al4V is questionable due to the toxic element Vanadium; however, nowadays, it is a common research material used in medical application. As found in current state-of-the-art orthopaedic total hip arthroplasty, the surface of the implants are very important for good osseointegration and to prevent possible infection; in addition to morphology, both the surface chemistry and biocompatibility of the materials are significant, which is why the research presented in this paper has a high possibility of real application.

An analysis of the state of the literature showed that the evaluation of additively produced surfaces is an important but still not fully described issue. The modelling of small morphological features with dimensions that are on the limits of fabrication possibilities and their subsequent evaluation is still a research gap and needs further investigation. Therefore, this article contains an evaluation of the surface textures of titanium femoral stem characterized by a certain distribution of irregularities, a porosity based on hemispheres of different sizes (which may have an impact on the future of the appropriate design of machines), 3D printers, and the introduction of correct process controls, taking into account the analysis of fabricated models with very small geometric features, especially for precision applications, such as medical use.

## 2. Scientific Context

### 2.1. Morphological Approach

Surface texturing is the technique of modifying a surface by creating certain morphological features on it. The placement of asperities, dimples, or channels at a specific location and of a specific size and shape is intended to improve the mechanical, performance, functional, or tribological properties of the components. Textured surfaces are widely used and every person—consciously or unconsciously, directly or indirectly—uses elements with such a texture. This occurs while driving a car, using non-slip pads, waiting at a pedestrian crossing, or using an elevator. The development of such surfaces with a specific distribution of irregularities and morphological features has its justification in connection with the physical and chemical phenomena that occur during the use of such surfaces. Textured surfaces are widely used in the automotive industry on pistons and cylinders, in thrust bearings, journal bearings, and MEMs, as well as in sealing. The use of such surfaces in sealing has beneficial effects on several factors such as increasing load-carrying capacity, fluid film stiffness, anti-seizer ability, and wetting behaviour and reducing wear, friction, leakage, and interface temperature [38]. A study focused on evaluating such surfaces in sealing aspects was conducted as early as 1996 by Etsion et al. In the study, it was shown that the use of hemispherical micro-dimples evenly distributed on one of the working planes results in an increase in the performance of such a seal [39]. This further results in a reduction in component temperature through better dissipation [40]. The feeding and discharge of working fluids and process components also has an important effect on cutting processes such as deep-hole drilling or threading. During such processes, it is difficult to supply the cutting fluid directly to the workpiece. Using surface texturing, the lubricant retained in the textures could help enhance the lubrication effects, thereby reducing the chance of tool breakage [41], as well as tool wear [42]. Studies [43] and [44] contain analyses of textured surfaces carried out for elements such as pistons and cylinders. This research was conducted in order to reduce unfavourable phenomena occurring during the interaction of these elements. This interaction involves operating these components at high temperatures, loads, and speeds, and a lack of lubrication can cause severe scuffing, seizure, and failure. Therefore, cross-hatched honing grooves are applied to cylinder liners to retain the lubricant, improve tribological properties, and extend the life of such components. The use of such grooves also undoubtedly has a beneficial effect on bearing operation, as it results in the generation of additional pressure resulting in a higher load-carrying capacity [45], as well as improved lubrication performance [46]. The creation of asperities, dimples, or channels is also extremely beneficial due to the increase in the real surface area of the elements produced in this way (extended surfaces). This has a beneficial effect on bonding and the nesting of textured material: for example, when applying an implant to tissue or bone [47]. Such microtextures enhance cell adhesion and perform an essential function in the elements’ contact [48].

### 2.2. Metrological Approach

Designing, fabricating, and then measuring a textured surface with a specific quality and distribution of individual morphological features means that the nominal, real, and measured surfaces are not identical. In each case, there are errors that affect the perception of such a surface [49,50]. There are technological limitations associated with the component fabrication process, such as layer thickness and minimum laser beam diameter, preventing the formation of small features on the surface. The difficulties associated with mapping a designed component using AM technology are related to a number of physical processes between the laser, the powder bed, and the layers underneath [51]. Moreover, when considering metrological aspects, it should be noted that real and measured surfaces are not the same due to a series of technological components affecting the measurement process. There are issues related to the problems of measurement of surfaces—in particular, surfaces fabricated using additive technologies, their evaluation including appropriate filtering of surfaces with specific morphological characteristics. It is important to consider both the shape and location of particular features in the studied area and the appropriate distance from the edge of the studied area [52] but also the choice of appropriate machines and methods for measuring such elements [53].

## 3. Materials and Methods

### 3.1. Sample Preparation

**Figure 2:**
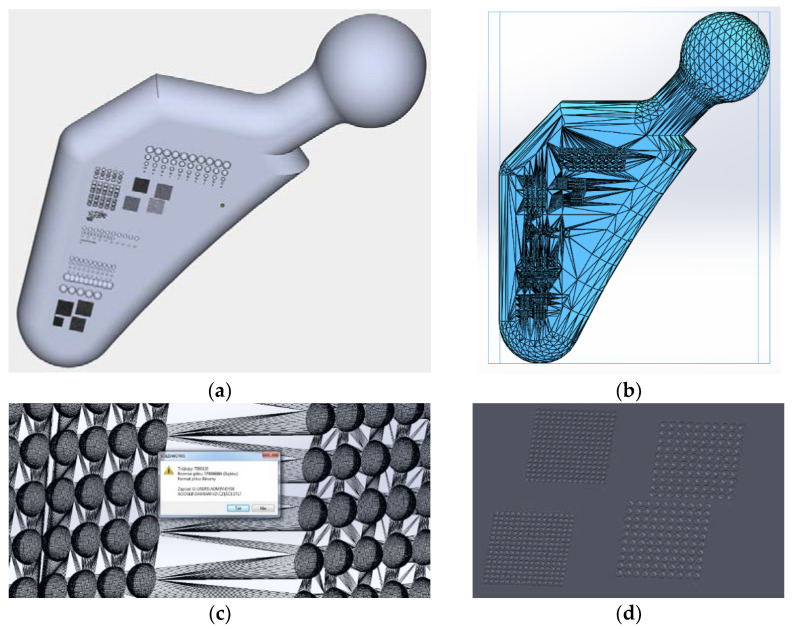
View of 3D models, CAD, (**a**) isometric view, (**b**) view of the saved .stl file, (**c**) view of the proposed saving of .stl files, too accurate, (**d**) magnified view of geometric features highlighted in red.

The test samples were designed as 3D solid models in SolidWorks (Dassault Systèmes SolidWorks Corp., Waltham, MA, USA). The design was made using available medical materials to replicate the actual hip implant stem model. The model was then saved as an .stl file using Magics software with the appropriate accuracy. The .stl file consisted of 424,550 triangles and had a linear accuracy combined with angular accuracy of +/−0.01 mm. The accuracy was selected in such a way so as to allow the .stl file to be processed by further 3D printer software. By writing the .stl with excessive accuracy, the machine’s internal software was unable to process the file further, so the accuracy remained at the presented level. Figure 2 presents a view of the CAD model and a view of the characteristic locations shown in Materialise Magics 23.0.0.289 (Leuven, Belgium).

### 3.2. Materials

A titanium-powder-based material with the trade name Ti6Al4V, produced by EOS (EOS GmbH, Krailling, Germany), was used to build the sample models. The chemical composition of the Ti6Al4V material and the mechanical properties claimed by the machine and material manufacturer are presented in Table 1 and Table 2 [54]. This material, in powder form, is characterised by a maximum particle size of 63 µm.

### 3.3. Method

**Figure 3:**
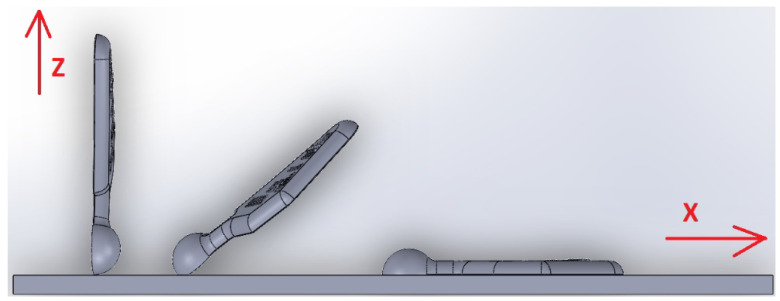
Visualization of 3D printer platform.

An EOS M290 machine (EOS GmbH, Krailling, Germany) was used to build the sample models. The test samples were made in quantities of three, where each sample was made by varying the orientation of the models on the build platform, which is shown in Figure 3. The samples were made with the following technological parameters: Inskin laser power—340 W, laser speed—1250 mm/s, hatch distance—0.12 mm, laser spot size—100 µm, layer thickness—60 µm. The platform temperature was set at a value of 35 °C, argon was used as a shielding, power fulfils ASTM F1472 and ASTM F2924 standards, and samples were heat treated at 800 °C for 2 h in argon inert atmosphere as instructed by EOS.

### 3.4. Measurement

The samples obtained by the method described above were subjected to the quality assessment of the geometric structure of the surface. The surface topography analysis was performed in two ways: surfaces with designed irregularities were evaluated as well as areas where such features were not present. It was considered crucial to compare the surface texture of the samples by using a classical approach based on the analysis of the primary profile, waviness, and roughness, allowing the evaluation of individual surface features. The tests were carried out using an optical profilometer Talysurf CCI Lite for which the principle of surface measurement is based on the method of coherent correlation interferometry (Miaru interferometer). A ×20 lens and stitching were used for measurements, obtaining a surface size of 1.51 × 1.51 mm, which is represented by a point matrix of 1844 × 1844. TalyMap Platinum 6 (Digital Surf, Besançon, France) software was used in the study.

### 3.5. Surface Topography Features

A key issue when producing morphological features on a surface is to determine the capabilities of the machine that is being used for this purpose. The limitations of the process will directly translate to the mapping capabilities of individual features, especially those with small sizes. Therefore, it was considered appropriate to evaluate the possibility of producing dimples and bumps in sizes close to the machine’s capabilities and layer thickness, as well as larger, when appropriate. Both convex and concave hemispheres with diameters equal to 0.04, 0.075, 0.11, and 0.15 mm were selected for analysis, each with a layer thickness of 0.06 mm. The hemispheres were made into a rectangular matrix in which the distance between the centres of the hemispheres was denoted by the parameter d, and the volume of one convex or concave hemisphere was denoted by the parameter V. The data are summarized in Table 3. Furthermore, in order to investigate the effect of print direction on the distribution of irregularities, a surface on which no morphological features were designed was also used for evaluation.

## 4. Results and Discussion

### 4.1. Surface Topography

**Figure 4:**
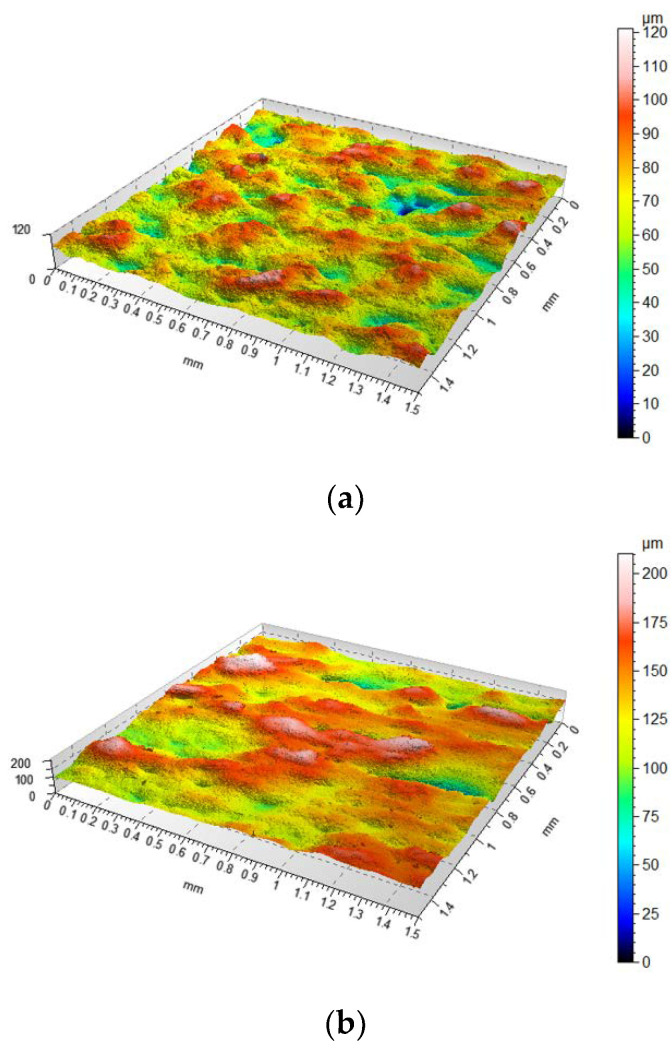

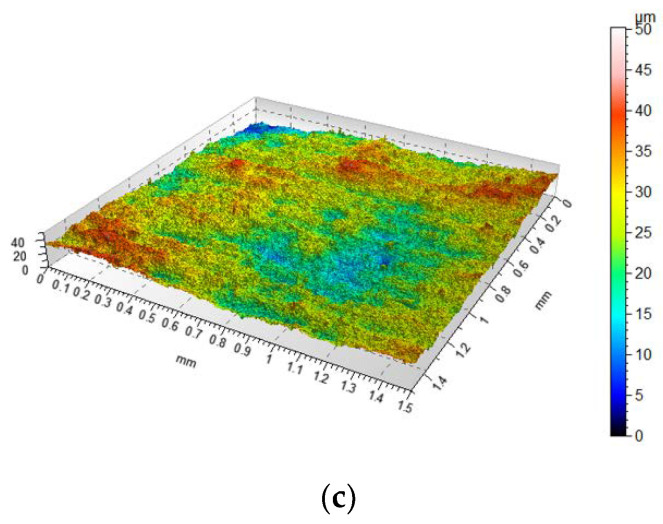
Example isometric view of a surface without modelled morphological features (**a**) 0° (**b**) 45° (**c**) 90°; scales should be analysed carefully.

The first stage of the research was the qualitative and quantitative evaluation of the surfaces created with the use of SLM technology, taking into account the angle in relation to the building plane. For this purpose, a number of parameters were selected to allow a quantitative description of the surface. Due to the nature of the morphological characteristics considered in the study, it was determined that the analysis of height and functional volume parameters was warranted. Both primary surface (P) and waviness (W) as well as surface roughness (R) were analysed. Example isometric views of the evaluated surfaces are presented in Figure 4 while parameter values for each surface are summarised in Table 4.

Upon the analysis of the obtained surfaces, it should be stated that there is a significant variation in the distribution and size of the irregularities depending on the orientation angle of the models on the platform during building. In Figure 4a, showing the surface obtained at 0°, it can be seen that the distribution of irregularities corresponds to a characteristic structure of pits and peaks whose height variation corresponds to the thickness of a single manufacturing layer. In individual areas, these irregularities are twice as large. This error is due to either the technological process of spreading the material on the build platform by the working arm of the machine or an improper sintering process. Figure 4b shows a similar distribution of irregularities, but in this case, a staircase effect is clearly visible, corresponding to successive parallel layers of material. The surface irregularities seen in Figure 4c are much smaller compared to the other surfaces. On the surface oriented vertically with respect to the build platform, the irregularities corresponding to the different layers of material can be noted, which is due to their size. In addition, one can also see the impact of the laser sintering process in places where the powder was not evenly distributed, resulting in irregularities. In this case, the qualitative analysis is confirmed by the obtained values of the parameters collected in Table 2. The surfaces are characterised by a similar distribution of irregularities, however, with different sizes of these irregularities. Both the parameter Ssk, being higher than 3 for almost all cases, and Sku, lower than 0, preserve a similar trend, reflecting the distribution of irregularities.

### 4.2. Topography of Surface Morphological Features

**Figure 5:**
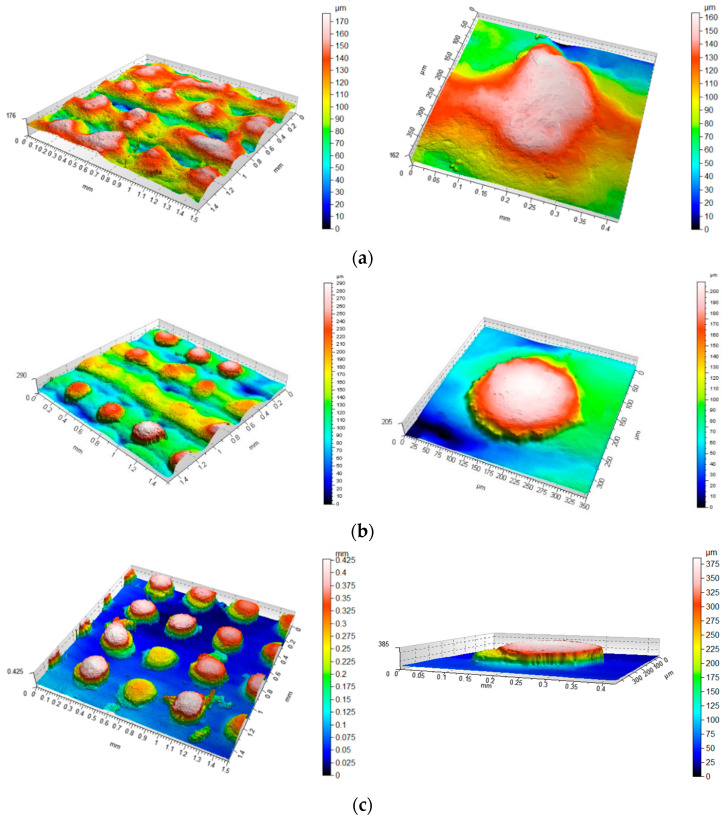
An isometric view of the surface of sample 1 together with a view of an example bump at an angle of (**a**) 0° (**b**) 45° (**c**) 90°; scales should be analysed carefully.

**Figure 6:**
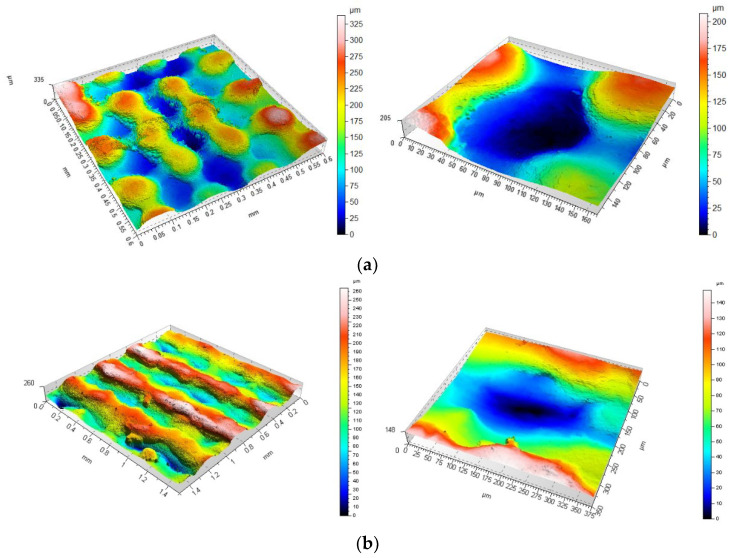

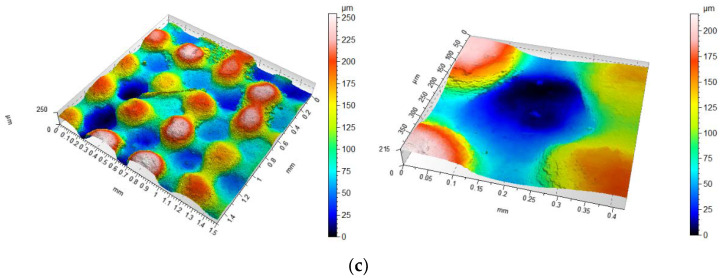
An example isometric view of the surface of sample 5 together with a view of an example dimple at an angle of (**a**) 0° (**b**) 45° (**c**) 90°; scales should be analysed carefully.

**Figure 7:**
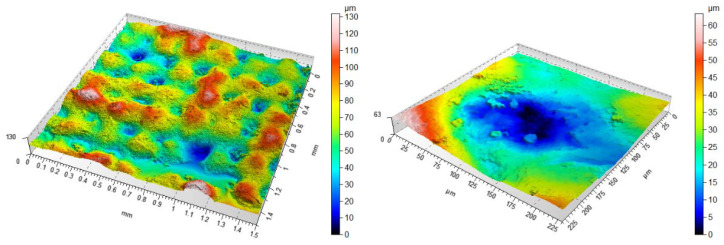
Example isometric view of the surface of sample 6 with a view of an example dimple at 90°; scales should be analysed carefully.

**Figure 8:**
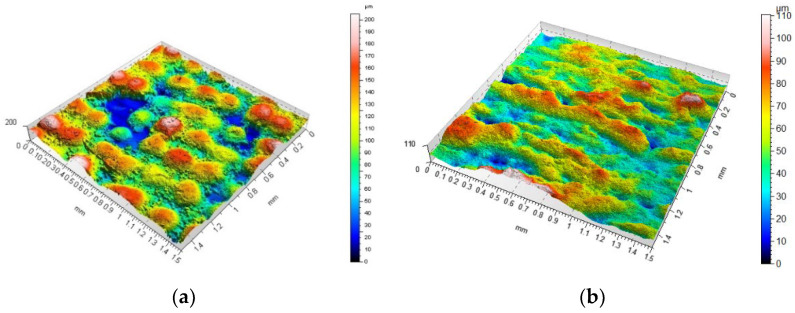

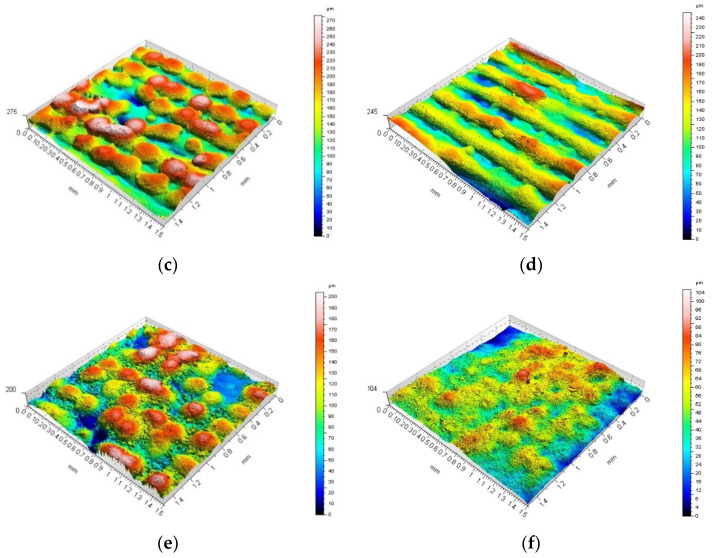
Examples of selected isometric views of samples taken at specific angles (**a**) sample 2—angle 0°, (**b**) sample 4—angle 0°, (**c**) sample 2—angle 45°, (**d**) sample 6—angle 45°, (**e**) sample 2—angle 90°, (**f**) sample 7—angle 90°; scales should be analysed carefully.

**Figure 9:**
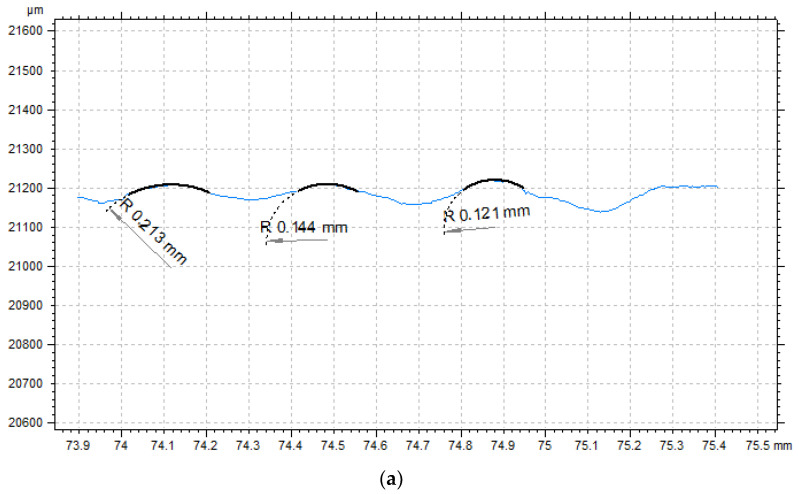

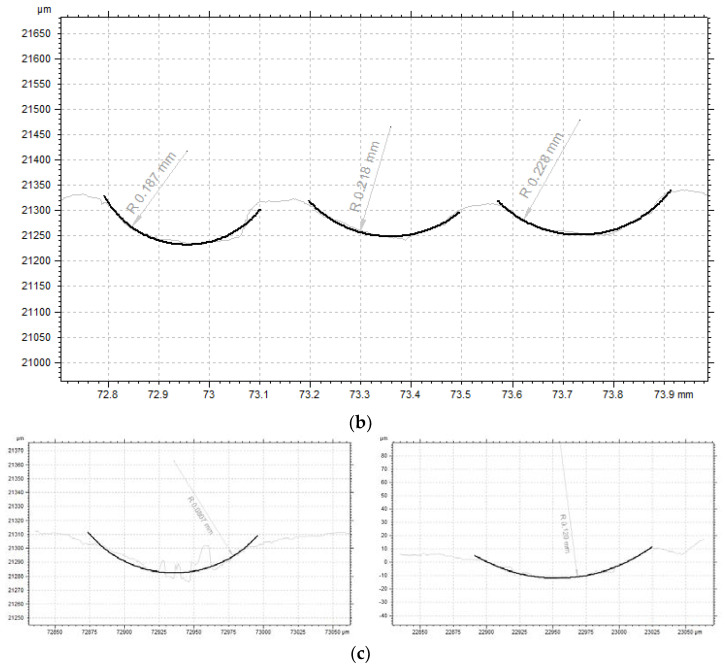
Example surface profile with defined values of the radius of morphological features (**a**) sample 1—angle 0° (**b**) sample 5—angle 90°, (**c**) sample 6—angle 90°.

The second key stage of the study was to evaluate the possibility of using SLM technology to produce relatively small (less than 0.3 mm) morphological features near the thickness of one layer. In this case, the orientation angle relative to the building plane is also considered. Modelled dimples and bumps of specific sizes were examined for their reproducibility on real models with real-life applications in the medical industry. Four values of the hemisphere radius were selected and the type of feature (convex/concave), as well as the model building angle, were taken into account, which allowed for a comprehensive evaluation of the feasibility of their fabrication along with the metrological analysis of such features. Figure 5, Figure 6, Figure 7, Figure 8 and Figure 9 show selected example images of surface features along with the specific radius values for each hemisphere. Example isometric images of the surface and its selected parts for sample 1 oriented at three different angles are presented in Figure 5.

In the figure above, the variation in the CAD model representation as a function of the building angle can be observed. There is a difference in both the size of the resulting morphological feature and its shape. An evaluation of the size of the resulting convex bumps shows that they were best mapped for a sample oriented at the angle of 0°. The difference between the designed hemisphere radius of 0.15 mm and the measurement result is small. What differs is the shape of the hemisphere. A “spillover” of material between the hemispheres can be observed. A surface produced at an angle of 90° looks entirely different. On this surface, individual surface features can be clearly seen, but their size is not the same as in CAD models. The diameters of the individual hemispheres are consistent with the model; however, the height varies—in most cases the hemispheres are more than twice as high (reaching 375 µm) as the model, and there is a characteristic flattening of the feature on the upper part, which may be related to the layer alignment process after sintering. The intermediate surface made at an angle of 45° represents the morphological features better. However, the different distribution of material on the two sides of the hemisphere can be observed on the surface. On one side, near the base, the increase in hemisphere height is abrupt, while on the opposite side, a much smoother change in height can be observed. This is due to the orientation of the model on the platform and the limitations of the technological process used to sinter the powder outside the assumed model (a typical error also given by printer manufacturers). The images also show a characteristic staircase effect.

Figure 6 shows isometric views for concave dimples. Both variations and similarities in the ability to produce convex and concave features can be observed. The magnitudes of the individual features correspond better to the CAD model; however, in this case, the effect of “spilling” material between hemispheres became apparent for all angles. In addition, the presence of formed material particles in the lower regions for concave dimples that were not designed in CAD was also noted in many cases. An example of an isometric image is shown in Figure 7. In order to better visualise the obtained surfaces, Figure 8 was prepared to present selected isometric views showing the variation of roughness for selected samples.

The considerable variation in irregularities and the reproducibility of the nominal features determined the need to evaluate the actual values of the radii of each hemisphere and its volume. Figure 9 shows examples of sample surface profiles with defined radius values for each morphological feature.

**Figure 10:**
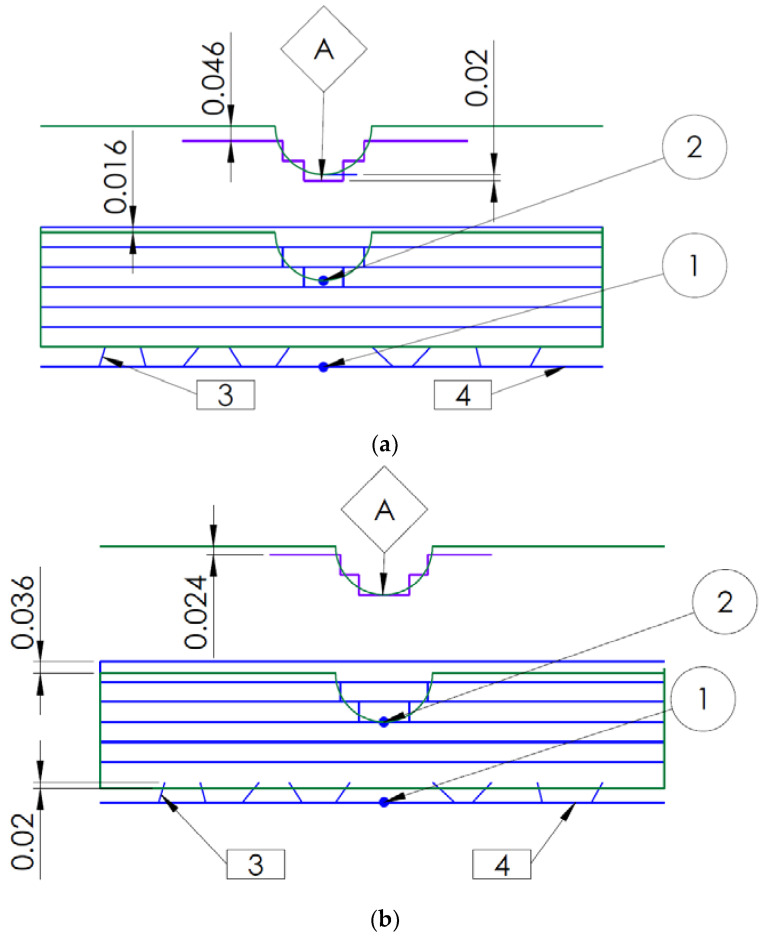
Process control: (**a**) standard type of basing, (**b**) improved type of basing according to the "dimple" module.

The values of the obtained radius of individual hemispheres vary depending on the fabrication angle and the location of the hemisphere on the surface, which was directly influenced by the fabrication process, i.e., its technological limitations and the process of material flow. The method of forming the material also directly contributed to changing the size of the actual surface, which is crucial from the point of view of implantology and nesting of the human material (living tissue) and the implant (printed object). The analysis of obtained values of the radius of particular hemispheres, performed on thirty different profiles obtained with different horizontal orientation, indicates that the obtained real values are close to the nominal ones; nevertheless, even in the area of one surface of the sample it is possible to notice the variation of such values, especially for radii with small values of the parameter. A similar assessment carried out for the volume (Table 5) of individual hemispheres showed that the values of the volume parameter were, in most cases, smaller than the nominal one; however, this difference is relatively small. This may be due to the melting process of the material, which caused the actual height of the hemisphere to be less than the nominal height. Evaluating the values obtained as a function of angle, it can also be noted that the staircase effect seen for the samples made at 45° resulted in the largest volume of features for these samples. Therefore, it can be concluded that such a surface can be reproduced in particular for radii higher than the thickness of two build layers. Results for four surfaces (45°—surface 4, 7, and 8 and 90°—surface 8) are not included in this table due to the fact that morphological features were not recorded in the indicated areas. In these areas, the distribution of surface roughness did not differ from the roughness in areas where hemispheres were not designed. The analysed areas, for which no volumetric morphological features were registered, were characterised by the lowest values of hemisphere radii, which implies that for the angle of 45° for orienting the models on the build platform, the fabrication of such features is impossible or is at the very limit of technological possibilities. It seems that the introduction of corrections in placement of models on the build platform in relation to the *z*-axis could significantly improve the quality of reproduced morphological features, which would require the introduction of additional modules responsible for the placement of models on the build platform, enabling a more complete process control. An example of such an improvement, one that would be made using adjustments to fabricate precise morphological features with small dimensions, is presented in Figure 10. The considerations noted above are confirmed by the obtained values of volumetric parameters. It can be noted that the values of the parameter determining the core material volume was the highest for the samples made at an angle of 45°. In addition, in order to better visualize the irregularities of the obtained surfaces, Table 6 was prepared, in which the values of selected 3D parameters for selected samples are presented.

### 4.3. Self-Decision Making Machine—Dimple Module

Figure 10 shows two variants of orientation with respect to the *z*-axis. The blue colour is used for the layers and the green colour for the CAD model. Designation 1 represents the standard type of basing to the base, while designation 2 is the reference point, the quadrant of the hemisphere. Reference 3 defines the supports, reference 4 is the base plane of the build platform, and point A defines a possible representation of the actual model. In Figure 10a, when orienting the model on a platform with supports underneath it for easy removal of the model after printing, the problem of the location of the quadrant of the hemisphere (reference point) arises. In this case, the reference point is located between the layers (20 µm above the bottom layer). This will make the model inaccurate—the structure (shape) of the hemisphere will not be preserved and the approximation effect of the .stl model will be disturbed. The solution to this problem is an additional “dimple” module whose main task in the process of orienting the model on the platform will be to place the reference point (the quadrant of the hemisphere, Figure 10b) in such a way that it is at the bottom of the currently built layer, which will positively affect the building process, the staircase effect, and the surfaces. Such an automated module, based on appropriate algorithms, would enable fast and efficient orientation of the model on the build platform and directly influence the process control. Such a module is a step on the way to building a self-decision-making machine which, after an analysis of the nature of irregularities, would adjust both the angle and the position of the model depending on the print parameters, in particular, the thickness of the built layer.

## 5. Partial Conclusions and Perspectives

The use of modern, unconventional fabrication methods such as 3D printing allows for the construction of functional elements with medical applications. These elements can have a certain degree of biocompatibility, which enhances their scope of application. This enables the printing of bone parts and implants and modelling specific porosity to ensure that they are connected with the living tissues of the patient. Such an evaluation will enable the manufacture of a model that will interface best with human tissue. In addition, the knowledge gained from the completed research will allow the selection of such parameters of the technological process of 3D printing to create surfaces with appropriate characteristics. This is an example of using the process-control phenomenon. An analysis of the results presented above led to the following general conclusions:

1. Depending on the orientation angle of the models on the build platform, process limitations relating to the inability to produce certain morphologic features were noted, particularly for the 45° angle. For each convex and concave hemisphere, different limits of features that could be produced on the surface were determined. The comparative evaluation of the ability to produce convex and concave hemispheres showed no significant differences between the types of morphological feature.
2. The individual hemispheres showed irregularities caused by errors in the fabrication process, i.e., forming irregular sintered powder particles in the lower areas of concave hemispheres, flattened tops in convex bumps, and material “spilling” between hemispheres. The surface texture of the samples without model hemispheres for the primary, waviness, and roughness parameters of the surface reached the largest values for the middle variant of orientation on the platform—45°. In addition, a staircase effect was clearly visible for this variant, which, in combination with the previous conclusion, places this orientation angle as the least favourable.
3. Analysis of the volume of real and designed features representing porosity showed that, for almost all cases, the obtained values of the parameter were lower than the nominal values. Only for convex dimples made at 45° were the values higher. It follows that a corresponding correction would have to be made in the CAD model.
4. For the analysis of clinical aspects, it seems that there is a key issue which can significantly affect the quality of models. The results can be applied to the manufacture of fully functional elements where the issue of tissue and element connection is extremely important. This involves the size of the extended surfaces of the elements. Dimples and bumps correspond to typical morphological surface features, and porosity is also a key factor in clinical applications. The main objective of the study was to assess such features in respect of clinical application.
5. Depending on the building angle, both for the surface on which the hemispheres were not designed and the surface with the characteristic system of features, a different distribution of surface roughness was noted. Individual roughness parameters varied, indicating the importance of accurate metrological control of real elements.
6. The obtained average values of the radii for the individual morphological features showed that it is possible to accurately represent them by a properly performed process control. Values close to nominal were obtained. However, depending on the sample, there was significant variation in the values depending on the size and type of the analysed feature.
7. The results of the study can be used as a guideline for the development of additional soft and hard modules for 3D printers, whose main goal would be to select the process-control parameters in such a way so that accurate models with specific characteristics could be fabricated using appropriate algorithms based on the direction of printing and height relative to the platform to build characteristic geometric features.

## Figures and Tables

**Table 1:** The chemical composition of the powder (wt, %).

Element	Al	V	O	N	C	H	Fe	Y	Other Elements, Each	Other Elements, Total	Ti
Value	5.5–6.75	3.5–4.5	0.2	0.05	0.08	0.015	0.30	0.005	0.1	0.4	Bal.

**Table 2:** Tensile data at room temperature.

Properties	Horizontal	Vertical
Ultimate tensile strength, Rm	1055 MPa	1075 MPa
Yield strength, Rp0.2	945 MPa	965 MPa
Elongation at break, A	13%	14%
Reduction of area, Z	>25%	>25%

**Table 3:** Summary of morphological features.

Sample Number	Type of Feature	R, mm	d, mm	Volume V, 10^−3^ mm^3^
1	convex	0.15	0.4	7.069
2	convex	0.11	0.25	2.788
3	convex	0.075	0.2	0.884
4	convex	0.04	0.13	0.134
5	concave	0.15	0.4	7.069
6	concave	0.11	0.25	2.788
7	concave	0.075	0.2	0.884
8	concave	0.04	0.13	0.134

**Table 4:** Values of selected 3D parameters.

	0°			45°			90°		
	P	W	R	P	W	R	P	W	R
Sq, µm	13.7	3.38	12.3	23.4	10.2	18.2	5.81	3.80	3.26
Ssk	−0.292	−0.917	−0.172	−0.464	−0.0357	−0.637	−0.256	−0.39	−0.081
Sku	3.55	3.68	3.39	4.26	2.07	5.97	3.13	3.06	3.16
Sp, µm	49.2	6.49	45.2	72.2	20.9	54.8	24.5	8.08	17.2
Sv, µm	71.9	11.8	62.7	138	22.7	128	25.8	13.1	15.4
Sz, µm	121	18.3	108	210	43.5	183	50.2	21.2	32.6
Sa, µm	10.9	2.71	9.78	18.4	8.67	13.6	4.60	3.07	2.59
Vm, 10^−3^ mm^3^/mm^2^	0.577	9.12 × 10^−2^	0.56	0.972	0.301	0.909	0.229	0.172	0.146
Vv, mm^3^/mm^2^	0.0177	0.0037	0.0161	0.0297	0.014	0.0225	0.00765	0.00462	0.00427
Vmp, 10^−3^ mm^3^/mm^2^	0.577	9.12 × 10^−2^	0.56	0.972	0.301	0.909	0.229	0.172	0.146
Vmc, mm^3^/mm^2^	0.0121	0.00318	0.0109	0.0207	0.0107	0.0147	0.00525	0.00358	0.00294
Vvc, mm^3^/mm^2^	0.016	0.00319	0.0147	0.0267	0.0132	0.0202	0.00692	0.00414	0.00388
Vvv, 10^−3^ mm^3^/mm^2^	1.66	0.51	1.44	2.92	0.796	2.34	0.725	0.471	0.392

**Table 5:** Volume of morphological features formed.

	Nominal	0°	45°	90°
1	0.113	0.089	0.104	0.115
2	0.100	0.095	0.107	0.063
3	0.050	0.049	0.053	0.038
4	0.019	0.030	-	0.031
5	0.113	0.071	0.068	0.116
6	0.100	0.051	0.042	0.053
7	0.050	0.039	-	0.033
8	0.019	0.017	-	-

**Table 6:** Values of selected 3D parameters for selected samples.

	Sample 2			Sample 3			Sample 6		
	0°	45°	90°	0°	45°	90°	0°	45°	90°
Sq, µm	25.1	33.4	26.5	39	49.3	39.7	20.1	36.8	23.9
Ssk	−0.229	−0.057	0.104	−0.23	−0.213	−0.098	0.614	0.734	0.292
Sku	2.68	2.94	2.27	2.57	2.32	2.44	3.68	3.29	2.85
Sp, µm	83.1	125	72.9	102	113	95.8	78.4	155	79.8
Sv, µm	87.6	122	74.7	103	164	108	61.3	99.3	61
Sz, µm	171	247	148	205	277	204	140	255	141
Sa, µm	20.6	27.1	22.1	31.6	41.8	32.5	15.4	29.6	19
Vm, 10^−3^ mm^3^/mm^2^	0.681	1.59	0.979	1.43	1.55	1.25	1.49	1.82	1.17
Vv, mm^3^/mm^2^	0.0335	0.042	0.037	0.0503	0.0635	0.0547	0.028	0.0572	0.0348
Vmp, 10^−3^ mm^3^/mm^2^	0.681	1.59	0.919	1.43	1.55	1.25	1.49	1.82	1.17
Vmc, mm^3^/mm^2^	0.0242	0.0316	0.0259	0.0357	0.0515	0.0377	0.0167	0.0326	0.022
Vvc, mm^3^/mm^2^	0.0308	0.0385	0.0346	0.0452	0.059	0.0503	0.0261	0.055	0.0325
Vvv, 10^−3^ mm^3^/mm^2^	2.67	3.58	2.4	5.09	4.42	4.42	1.86	2.25	2.24

## Data Availability

The data presented in this study are available on request from the corresponding author.
